# CALCOCO2/NDP52 initiates selective autophagy through recruitment of ULK and TBK1 kinase complexes

**DOI:** 10.1080/15548627.2019.1628548

**Published:** 2019-07-01

**Authors:** Keith B. Boyle, Benjamin J. Ravenhill, Felix Randow

**Affiliations:** a Division of Protein and Nucleic Acid Chemistry, MRC Laboratory of Molecular Biology, Cambridge, UK; b Department of Medicine, Addenbrooke’s Hospital, University of Cambridge, Cambridge, UK

**Keywords:** Autophagy, cargo receptor, eat-me signal, FIP200, galectin-8, NDP52, Salmonella, TBK1, ULK1

## Abstract

The selective macroautophagy of prospective cargo necessitates activity of the autophagy machinery at cargo-determined locations. Whether phagophore membranes are recruited to, or are generated *de novo* at, the cargo is unknown. In our recent study we show that damaged *Salmonella*-containing vacuoles, marked by LGALS8/galectin-8, engage the cargo receptor CALCOCO2/NDP52 to recruit the autophagy-initiating ULK and TBK1 complexes and cause the formation of WIPI2-positive phagophore membranes. CALCOCO2 functions in the induction of autophagy by forming a trimer with RB1CC1/FIP200 and TBKBP1/SINTBAD-AZI2/NAP1, components of the ULK and TBK1 kinase complexes, respectively. Such recruitment of the upstream autophagy machinery to prospective cargo reveals how in complex eukaryotes detection of cargo-associated ‘eat me’ signals, induction of autophagy, and juxtaposition of cargo and phagophores are integrated.

Eukaryotic cells remove unwanted cellular components as well as invading pathogens by selective autophagy. Whereas encapsulation of the designated cargo inside an autophagosome is a defined end point of this process, it is unclear as to whether phagophores are generated *de novo* at the cargo location or whether pre-existing phagophores are recruited from elsewhere in the cell. Importantly, phagophore formation *in situ* may require recruitment of the autophagy initiating ULK complex to prospective cargo. In order to investigate the earliest steps of selective autophagy we took advantage of anti-bacterial autophagy, xenophagy, because the distinct nature and relatively large size of bacteria should benefit microscopy-based approaches in particular. Previous work from our laboratory had revealed that xenophagy of *Salmonella enterica* serovar Typhimurium shortly after rupture of the *Salmonella-*containing vacuole (SCV) is uniquely dependent on the cargo receptor CALCOCO2. In contrast, later during infection, when polyubiquitin has accumulated on cytosolic bacteria, other cargo receptors, including SQSTM1/p62 and OPTN (optineurin), also contribute. The unique and non-redundant role of CALCOCO2 in early xenophagy relies on binding of CALCOCO2 to the ‘eat-me’ signal LGALS8 that rapidly accumulates on those damaged SCV membranes. We now utilize anti-bacterial autophagy to show that the ULK complex, itself necessary to restrict the proliferation of cytosolic *Salmonella*, is recruited to damaged SCVs in a manner dependent on CALCOCO2 and LGALS8 (Figure 1) [1]. By means of yeast two-hybrid technology we identified the ULK-subunit RB1CC1 as a novel CALCOCO2-interacting protein and demonstrated that not only does CALCOCO2 thereby recruit the ULK complex but that it concomitantly interacts with the essential xenophagy kinase TBK1 via its adaptor proteins TBKBP1 and AZI2. Formation of the CALCOCO2-ULK-TBK1 super-complex is essential for xenophagy; specific abrogation of the CALCOCO2 interaction with either ULK or TBK1, by mutation of selective residues in CALCOCO2, results in the failure to form WIPI-positive phagophores and LC3-positive autophagosomes. Residues on opposite faces of the SKICH domain of CALCOCO2 are essential for binding to RB1CC1 and TBKBP1-AZI2, respectively, with hydrophobic patches centered around Tyr70 required for the former, and Ala119 for the latter.

**Figure 1.**
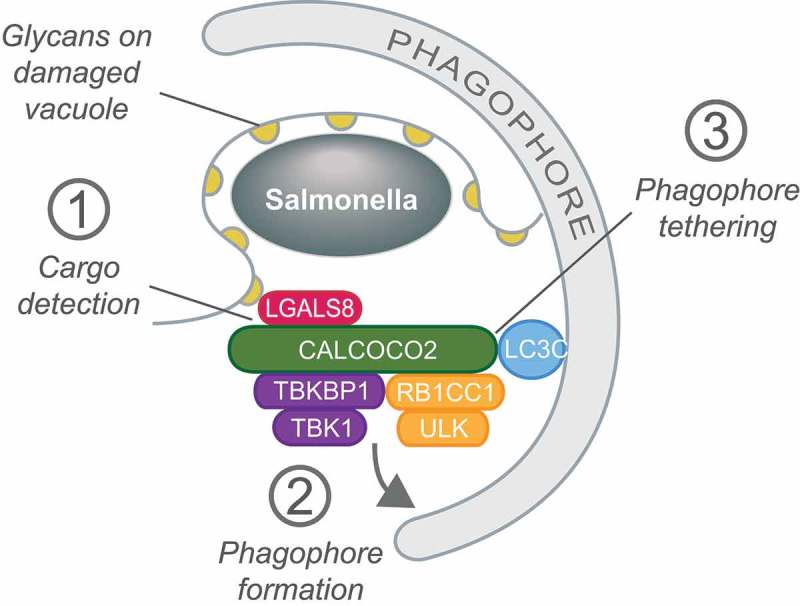
CALCOCO2/NDP52 orchestrates xenophagy by (1) detecting the ‘eat-me’ ligand LGALS8/galectin-8 on damaged SCVs, (2) initiating phagophore membrane formation by recruiting the ULK and TBK1 kinase complexes and (3) tethering damaged SCVs to the phagophore via LC3C.

RB1CC1 is the closest mammalian ortholog of yeast Atg11, known to integrate multiple selective autophagy pathways in yeast by binding characteristic di-aliphatic motifs in specific cargo receptors, e.g. Atg30 in mitophagy and Atg36 in pexophagy. We found that RB1CC1 binds a similar dialiphatic ‘IL’ motif not in CALCOCO2 itself but in TBKBP1 and AZI2, which we will refer to as a FIP200-interacting region. Indeed, RB1CC1 was recently shown to engage the reticulophagy cargo receptor CCPG1 via a related FIP200-interacting region motif. We therefore conclude that RB1CC1 conserves the function of Atg11 to engage cargo receptors with the core autophagy machinery. Whereas the direct interaction of RB1CC1 with TBKBP1-AZI2 was not required for the xenophagy of *Salmonella*, due to the dominant role of CALCOCO2 in this system, we were able to characterise the FIP200-interacting region-binding residues of RB1CC1 (N1572, F1574, V1576). The interaction of these residues with dialiphatic FIP200-interacting region motifs in other cargo receptors, or their adaptors, may play a more important role in the selective autophagy of other cargo.

Having found that LGALS8, CALCOCO2 and RB1CC1 were necessary for recruitment of WIPI and LC3 to damaged SCVs, we next deployed structured illumination microscopy, a super-resolution technique, to precisely locate each of these proteins. Whereas LGALS8, CALCOCO2 and RB1CC1 colocalize on the damaged SCV membrane, the phagophore-associated protein WIPI2 forms striking, donut-shaped structures, reminiscent of omegasomes, in the immediate bacterial vicinity distinct from the former proteins. We propose that these WIPI2 structures represent multiple phagophores around the bacteria, which may subsequently fuse, thereby increasing the likelihood that the bacterium becomes encapsulated in an autophagosome and its replication is thwarted.

An obvious question arising from our work is what controls the precise location of the WIPI2-positive omegasomes in the bacterial vicinity. We speculate that recruitment of the PI3KC3/VPS34 complex will be necessary for the generation of the WIPI2 ligand phosphatidylinositol-3-phosphate, which provides ample opportunity for further investigation. On a more general note, the topological conundrum of how exposure of the glycan ligand on the damaged SCV membrane does not solely result in phagophore engulfment of that membrane but also includes the associated bacterium still stands. One cannot dismiss the possibility that in addition to the LGALS8 signal on the damaged SCV, another ligand(s) on the bacteria, detected by as yet unidentified host factors, may help direct phagophore formation at the foreign invader. Further work, in particular using super-resolution microscopy that proved so insightful in revealing the arrangement of CALCOCO2, RB1CC1 and WIPI2 at cargo, will hopefully provide answers to these questions.

We posit that the CALCOCO2-dependent engagement of selective autophagy upon bacteria-mediated vacuolar damage is a neat experimental system, devoid of cargo receptor redundancy, to interrogate further how selective autophagy is accomplished. The primacy of CALCOCO2 in xenophagy has permitted several important functions to be attributed to this protein: i) the detection of the ‘eat-me’ signal LGALS8 on damaged SCVs, ii) the initiation of phagophore formation through recruitment of the ULK and TBK1 complexes, iii) the tethering of cargo to phagophore membranes via LC3C, and iv) autophagosomal maturation via MYO6 (myosin VI). It will be interesting to see whether CALCOCO2 harbors yet other unidentified autophagy functions and whether other cargo receptors similarly engage the selective autophagy pathway at multiple junctures.

## References

[1] Ravenhill BJ, Boyle KB, von Muhlinen N, et al The cargo receptor NDP52 initiates selective autophagy by recruiting the ULK complex to cytosol-invading bacteria. Mol Cell. 2019 4 18;74(2):320–329. 30853402 10.1016/j.molcel.2019.01.041 PMC6477152

